# Healthcare costs and mortality associated with serious fluoroquinolone‐related adverse reactions

**DOI:** 10.1002/prp2.931

**Published:** 2022-02-16

**Authors:** Laura S.M. Kuula, Janne T. Backman, Marja L. Blom

**Affiliations:** ^1^ Faculty of Pharmacy University of Helsinki Helsinki Finland; ^2^ Individualized Drug Therapy Research Program Faculty of Medicine University of Helsinki Helsinki Finland; ^3^ Department of Clinical Pharmacology University of Helsinki and Helsinki University Hospital Helsinki Finland

**Keywords:** adverse drug reactions, antibiotics, costs, decision analysis

## Abstract

The aim of this study was to estimate healthcare costs and mortality associated with serious fluoroquinolone‐related adverse reactions in Finland from 2008 to 2019. Serious adverse reaction types were identified from the Finnish Pharmaceutical Insurance Pool’s pharmaceutical injury claims and the Finnish Medicines Agency’s Adverse Reaction Register. A decision tree model was built to predict costs and mortality associated with serious adverse drug reactions (ADR). Severe *clostridioides difficile* infections, severe cutaneous adverse reactions, tendon ruptures, aortic ruptures, and liver injuries were included as serious adverse drug reactions in the model. Direct healthcare costs of a serious ADR were based on the number of reimbursed fluoroquinolone prescriptions from the Social Insurance Institution of Finland’s database. Sensitivity analyses were conducted to address parameter uncertainty. A total of 1 831 537 fluoroquinolone prescriptions were filled between 2008 and 2019 in Finland, with prescription numbers declining 40% in recent years. Serious ADRs associated with fluoroquinolones lead to estimated direct healthcare costs of 501 938 402 €, including 11 405 ADRs and 3,884 deaths between 2008 and 2019. The average mortality risk associated with the use of fluoroquinolones was 0.21%. Severe *clostridioides difficile* infections were the most frequent, fatal, and costly serious ADRs associated with the use of fluoroquinolones. Although fluoroquinolones continue to be generally well‐tolerated antimicrobials, serious adverse reactions cause long‐term impairment to patients and high healthcare costs. Therefore, the risks and benefits should be weighed carefully in antibiotic prescription policies, as well as with individual patients.

## Graphical abstract

In this present study 1 831 537 FQ prescriptions filled between 2008 and 2019 were estimated to trigger 11 405 ADRs and 3,884 deaths, resulting in a total healthcare cost of 501 938 402 € during the twelve‐year observation period. The most impactful of the FQ‐related ADRs are severe *clostridioides difficile* infections (SCDI), which alone cause more than 95% of the total direct healthcare costs of the ADRs. Accordingly, from the economical perspective, efforts should be focused on limiting the risk of FQ‐associated CDI.

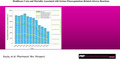

**Abbreviations**
ADR: adverse drug reaction
CDI: *clostridioides difficile* infection
DILI: drug induced liver injury
FQ: fluoroquinolone
IC: insurance claim
LOS: length of stay
SCAR: severe cutaneous adverse reaction
SCDI: severe *clostridioides difficile* infection
SJS: Stevens‐Johnson syndrome
TEN: toxic epidermal necrolysis

## 1 INTRODUCTION

Fluoroquinolones (FQs) are a group of antimicrobial drugs, which have activity against gram‐positive and gram‐negative bacteria and are used to treat genitourinary, respiratory, gastrointestinal, skin, and soft tissue infections. FQs have been in wide clinical use for over 30 years. In Finland, the number of FQ prescriptions has steadily remained at 5–6% of all antibiotic prescriptions.^1^ Although FQs are still considered to be mostly well tolerated, there have been increasing concerns about their safety. Their most common adverse reactions are mild and reversible, such as diarrhea, nausea, or headaches, and lead to termination of treatment in less than two percent of patients. However, fluoroquinolones are also associated with serious adverse drug reactions, such as recurring *clostridioides difficile* infections (CDI), cardiovascular toxicity, musculoskeletal, renal, and liver disorders, and reactions involving the central nervous system.^2^ Although the mechanisms behind FQ‐related adverse effects remain unclear, for example, mitochondrial damage has been implicated in the process.^3^, ^4^

Serious adverse reactions are defined as events that either result in death, are life‐threatening, require inpatient hospitalization or prolong current hospitalization, or result in persistent or significant disability or incapacity.^5^ Due to reports of serious ADRs associated with the use of FQs, the European Medicines Agency has recommended restrictions on their use several times.^6^ Although the total burden of FQ‐related ADRs has previously not been calculated, ADRs are generally recognized as an important cause of morbidity and even death. For example, according to Finnish studies, about 8% of hospital emergency visits and 3%–5% of in‐hospital deaths are explained by ADRs, corresponding to approximately 0.05% of all hospital admissions.^7^, ^8^, ^9^ Apart from direct health consequences, estimates of the economic burden of unsafe care due to ADRs have varied between 0.2% and 6.0% of the total health expenditure.^10^ The impact of the ongoing COVID‐19 pandemic on FQ‐related ADRs is still unknown. For example, according to a systematic review from 2021, 1% of COVID‐19 patients were reported to have *clostridioides difficile* infections (CDI).^11^ However, the authors conclude that there is little change in the overall incidence of CDI compared to the time before the pandemic.

While it is important to understand the economic burden of ADRs, research of the costs is challenging since most of them remain unreported.^12^ The findings of a systematic review from 2016 propose that published literature does not provide sufficient information on ADRs and that the majority of information is found in ‘grey literature’, such as government reports, working papers, press releases, theses, and conference proceedings.^13^ The aim of this study was to estimate healthcare costs and risk of mortality associated with serious FQ‐related adverse reactions in Finland between 2008 and 2019 using registry‐based and literature data and a decision tree modeling approach.

## 2 MATERIALS AND METHODS

### 2.1 Model parameters and sources of data

Serious FQ‐related adverse reaction types were identified from the Finnish Pharmaceutical Insurance Pool’s pharmaceutical injury claims and the Finnish Medicine Agency’s Adverse Reaction Register, which were available from 2002 to 2012, except for aortic ruptures, which were yet to be recognized as FQ‐related ADRs. The probabilities of serious ADRs were estimated based on published literature and are depicted in Table 1. When possible, literature sources similar to the Finnish population were preferred. FQs in use in Finland between 2008 and 2019 were included in the model. Tendon injuries, severe *clostridioides difficile* infections (SCDI), severe cutaneous adverse reactions (SCAR), acute liver injuries, and aortic ruptures were included as serious ADRs in the model. The overall incidence of FQ‐related serious ADRs is estimated to be between 0.1% and 1%.^14^ The model was developed according to the good practice guidelines and checklist for modeling in health technology assessment suggested by Philips et.al.^15^

**TABLE 1:** Model parameters and sources of data to estimate costs and mortality associated with serious adverse reactions in Finland in years 2008 to 2019

ADR type	Model parameters	Base case		Sensitivity analysis		Sensitivity analysis	
		Value	Reference	Lower	Reference	Upper	Reference
	FQ Prescriptions in 2008–2019 (n)	1,831,537	KELA				
	The cumulative risk of Serious ADR	0.0062		0.0031		0.033	
SCAR	LOS, range (n)	3–14	IC,^44^				
	Clinic visits (n)	N/A					
	ADR Risk	0.00001	[33]	0.000001	[34]	0.00008	[45]
	Mortality Risk	0.2	[46]	0.1	[33]	0.49	[34]
SCDI	LOS, range (n)	11–53	IC				
	Clinic visits (n)	N/A	IC				
	ADR Risk	0.0055	[16, 17]	0.0028	[16, 18]	0.0074	[16, 19]
	Mortality Risk	0.35	[47]	0.30	[24]	0.57	[48]
Tendon rupture	LOS, range (n)	15–25	IC				
	Clinic visits (n)	4	IC				
	ADR Risk	0.00029	[49]	0.00004	[50]	0.021	[42]
	Mortality Risk	0		0		0	
Liver injury	LOS, range (n)	8–15	IC				
	Clinic visits (n)	3	IC				
	ADR Risk	0.00023	[51]	0.000106	[51]	0.0011	[52]
	Mortality Risk	0.50	[53]	0.23	[54]	0.61	[38]
Aortic rupture	LOS (n)	9	[55]				
	Clinic visits (n)	1–15	[56]				
	ADR Risk	0.00018	[41]	0.00018	[41]	0.00357	[42]
	Mortality Risk	0.41	[56]	0.33	[43]	0.72	[42]

Abbreviation

IC, Insurance claims, 2001–2011; KELA, Social Insurance Institution of Finland; Risk, absolute risk; LOS, length of stay (hospital days); SCAR, Severe cutaneous adverse reaction.

Estimates of direct healthcare costs associated with serious FQ‐related adverse effects in Finland of a 12‐year period from 2008 to 2019 were based on the number of reimbursed FQ prescriptions collected from the Social Insurance Institution of Finland’s database. The numbers of prescriptions were multiplied by the probability of a serious ADR and the estimated cost of a single serious FQ‐related ADR episode. Mortality estimates were calculated by multiplying the estimated risk of mortality due to ADR with the number of reimbursed FQ prescriptions. Since there was no valid data source on the risk of FQ‐related SCDI, our estimates combined an overall risk of FQ‐related CDI based on the study from Despande et al. (0.092)^16^ with the risk of developing a severe infection, based on the studies from Zaiβ et al. (base case 0.06), Adams et al. (lower estimate 0.03) and Li et al. (higher estimate 0.08)^17^, ^18^, ^19^ All estimates were rounded. FQs prescribed in the study comprised ciprofloxacin, levofloxacin, moxifloxacin, ofloxacin, and norfloxacin.

### 2.2 Severe clostridioides difficile infections

*Clostridioides difficile* bacteria are members of normal gut microflora that are resistant to numerous antimicrobials and can thus colonize the human gut after antimicrobials have altered the proportions of the microbiota. The toxin expression of the bacteria results in gastrointestinal illness.^20^ The overall incidence of CDI after the use of FQs is estimated to be between 3 and 10 percent.^16^, ^20^ Since the emergence of the epidemic *Clostridioides difficile* ribotype 027 clone, CDI has become more prevalent, severe, and more difficult to treat due to resistance to many antimicrobial agents.^21^ The clinical course of the disease can range from asymptomatic colonization or mild diarrhea to severe infections characterized by pseudomembranous colitis, toxic megacolon, colonic perforation, or death.^20^ Advancing age and a history of partial colostomy are among the risk factors associated with more severe disease.^22^ The treatment options of CDI include vancomycin, metronidazole, fidaxomicin, fecal microbiota transplants, and, in life‐threatening cases, the surgical management of CDI complications.^23^ Severe CDI (SCDI) increases the risk of mortality to 57%.^24^ Additionally, patients over 80 years and those with increased inflammatory parameters and diagnosed sepsis have an increased risk of mortality.^25^ Previous research suggests that CDIs are associated with the highest costs among FQ‐related ADRs, although published literature remains scarce.^26^ According to the Finnish National Infectious Diseases Register, the overall number of CDI cases has reduced by 60% since 2008.^27^

### 2.3 Tendon ruptures

FQs were first connected to tendon injuries in 1983^28^ and these ADRs remain the most distinctive and reported FQ ADRs to this day. The incidence of fluoroquinolone‐related tendon injuries has been estimated to average 15–20 per 100 000 patients. The risk of tendon injuries is higher with over 60‐year‐old people, in particular males, and patients on corticosteroid therapy.^29^ Regardless of relieving pressure and avoiding activities that strain the tendon, fluoroquinolone‐related tendon injuries may result in tendon ruptures, which are treated with immobilization and/or corrective surgery.^30^ Although tendon ruptures might be risk factors for the physical and psychological decline, there is no evidence of them being a direct cause of death.

### 2.4 Severe cutaneous adverse reactions

Hypersensitivity to FQs can cause rare severe cutaneous adverse reactions (SCAR), which can be life‐threatening and is often preceded by hypersensitivity to beta‐lactams.^31^ Stevens‐Johnson syndrome (SJS) and toxic epidermal necrolysis (TEN) represent a spectrum of severe reactions varying with respect to the proportion of skin affected. SJS affects less than ten percent of the skin, whereas TEN is more serious and involves more than 30 percent of the body surface area.^32^ Overlapping of SJS/TEN is seen in 5% of cases. The mortality rates associated with SJS and TEN are between 10 and nearly 50 percent.^33^, ^34^ SCARs are commonly treated with intravenous immune globulin and corticosteroids and always require hospitalization. Variations in the HLA‐B gene are suggested to influence the occurrence of SCARs.^35^

### 2.5 Acute liver injuries

FQ‐associated hepatotoxicity ranges from mild transient aminotransferase elevations to hepatitis and jaundice and even to severe and acute liver injuries and hepatic failure. The onset may vary and, similarly to SCAR, possibly occurs via immunological mechanisms.^36^, ^37^ Acute FQ‐related liver injuries are generally rare, with a rate per 100 000 exposures of approximately 6 to 9 for ciprofloxacin, moxifloxacin, and levofloxacin.^38^ Identifying and unequivocally diagnosing drug‐induced liver injury (DILI) is difficult. Symptoms, such as abdominal pain, fever, nausea, vomiting, diarrhea, or rash are unspecific and patients can have prior liver damage from alcohol consumption, polypharmacy, or underlying liver disease, which further complicate the diagnosis.^39^ Yet, mortality associated with severe liver injuries is known to be high.^38^ The use of corticosteroids may be beneficial, although long‐term data for the treatment of DILI is still lacking.^40^

### 2.6 Aortic dissection and rupture

The array of FQ‐associated cardiovascular toxicity has amplified in recent years. QT interval prolongation and torsade de pointes in connection with FQs were described in the 1990s in several publications. Additionally, the systemic use of FQs is now known to increase the risk of aortic aneurysms and dissection,^41^ potentially leading to fatal hemorrhage. Together with tendon injuries, aortic ruptures have been linked to FQ‐related collagen toxicity, which may also be involved in FQ‐associated retinal detachment.^42^ According to estimates of a Finnish study, almost half of the patients with ruptured aneurysms die before they reach the hospital. Mortality associated with emergency surgery of aortic ruptures is 33%.^43^

### 2.7 Health service use and costs

The study was conducted from the perspective of the healthcare provider and addresses direct healthcare costs. Outpatient visits and hospitalizations were valued with the unit costs of non‐psychiatric specialized healthcare in Finland and contained all costing items related to the health service use, including laboratory tests, dispensed drugs, technical aids, and medical imaging costs. These costs did not include healthcare client fees, which equates to approximately 5 percent of healthcare costs.^57^ All costs were converted to 2019 euro. Missing health service use data were replaced by values from published literature. Since direct healthcare costs did not accrue past the first year, there was no need to discount costs.^58^

### 2.8 Modeling approach to adverse reactions

The decision tree is a commonly used tool for decision analysis in healthcare decision‐making and economic evaluations of health technologies.^59^ The decision tree model is suitable for modeling events that last for a limited period of time and generally do not involve relapses. Modeling techniques allow the synthesizing of evidence. The model pathways or branches are preceded by decision nodes and chance nodes and accompanied by probabilities of a particular outcome occurring at chance nodes. The decision tree model was built with TreePlan software for Microsoft Office Excel in order to estimate costs and mortality associated with serious ADRs.

Modeling is a simplification of reality and, thus, entails certain assumptions:

- FQs are not distinguished from one another in the model as their ADR profile is assumed to be similar
- All patients were hospitalized due to serious ADRs and required additional health services
- Absolute risks were drawn from literature and assumed similar, although they may vary in different countries due to genetic factors, study designs, and healthcare systems. Additional confounders in the model are possible comorbidities, polypharmacy, duration, and dose of FQs, and use of multiple antibiotics
- Data for serious ADR costs and health service use were available from 2002 to 2012 and it was assumed that treatment guidelines matched the FQ‐prescription period from 2008 to 2019.

### 2.9 Sensitivity analyses

Due to varying estimates of FQ‐related ADR and mortality incidence, the decision tree model contains uncertainty about the exact probabilities used as inputs. To deal with parameter uncertainty in the model, sensitivity analyses were conducted to assess their impact on direct healthcare costs and ADR‐related mortality.^60^ The lowest probability for an ADR and mortality generated the best‐ and highest probability yielded the worst‐case scenario, respectively.

## 3 RESULTS

**FIGURE 1:**
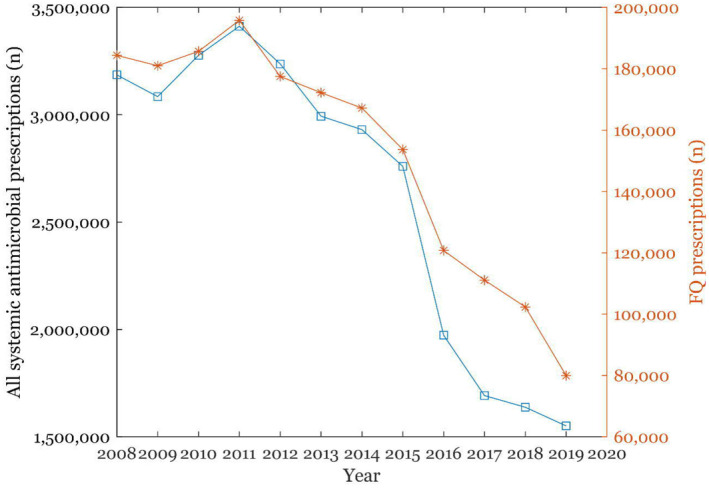
The annual number of fluoroquinolone prescriptions and all systemic antimicrobial prescriptions filled during the years 2008–2019 in Finland

A total of 1 831 537 FQ prescriptions were filled between 2008 and 2019. During the twelve‐year observation period, the number of FQ prescriptions fell by 40% from 2008 (n = 184 369) to 2019 (n = 79 926), peaking at 195 806 in 2011. In Finland, the number of FQ prescriptions has declined consistently with all antimicrobial prescribing (Figure 1).

**FIGURE 2:**
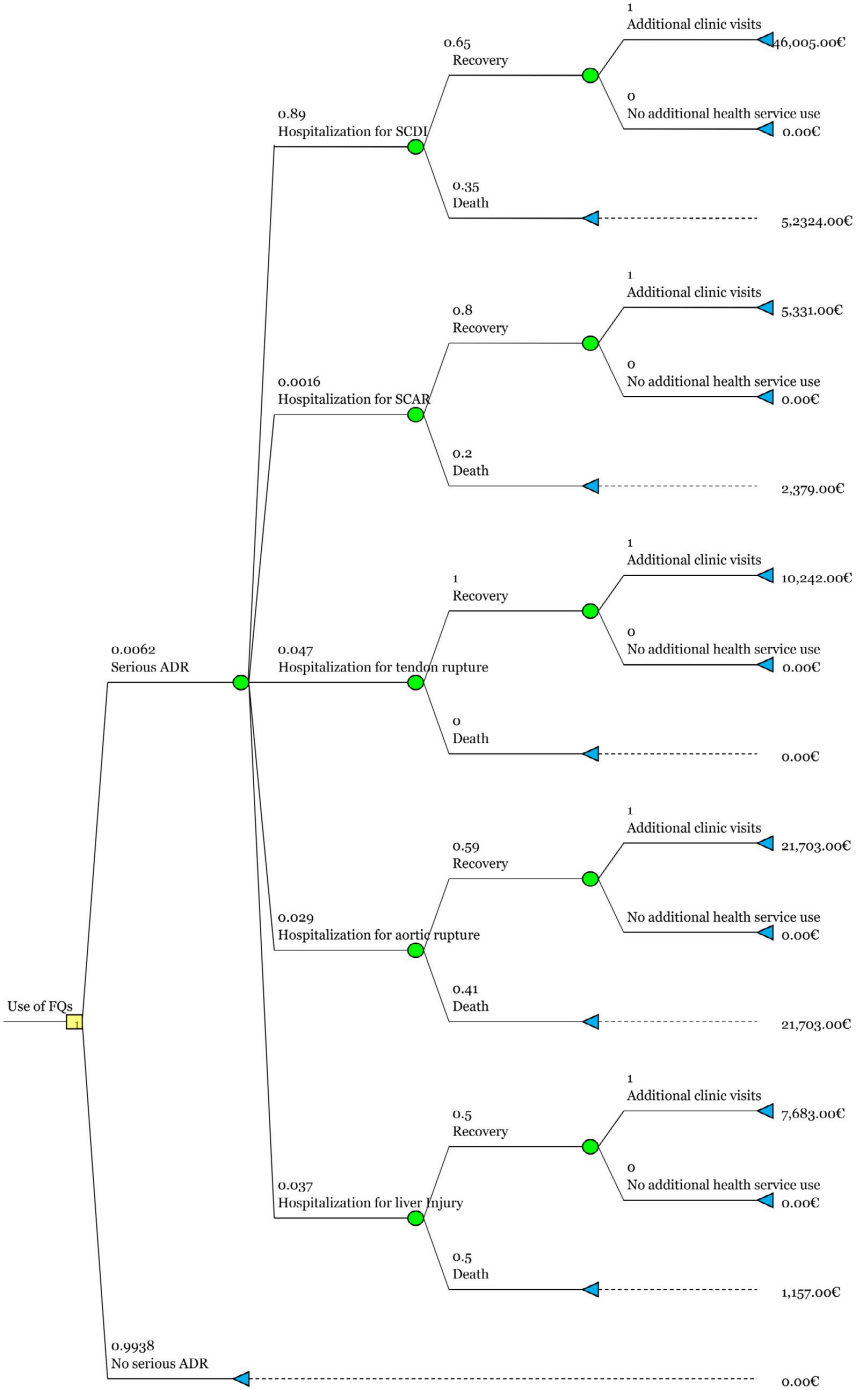
The structure of the decision tree estimating annual costs and mortality associated with serious FQ‐related ADRs. The model begins at the left at the square decision‐making node. Probability nodes are circular and amount to 1. The triangular terminal node describes the cost outcome

**FIGURE 3:**
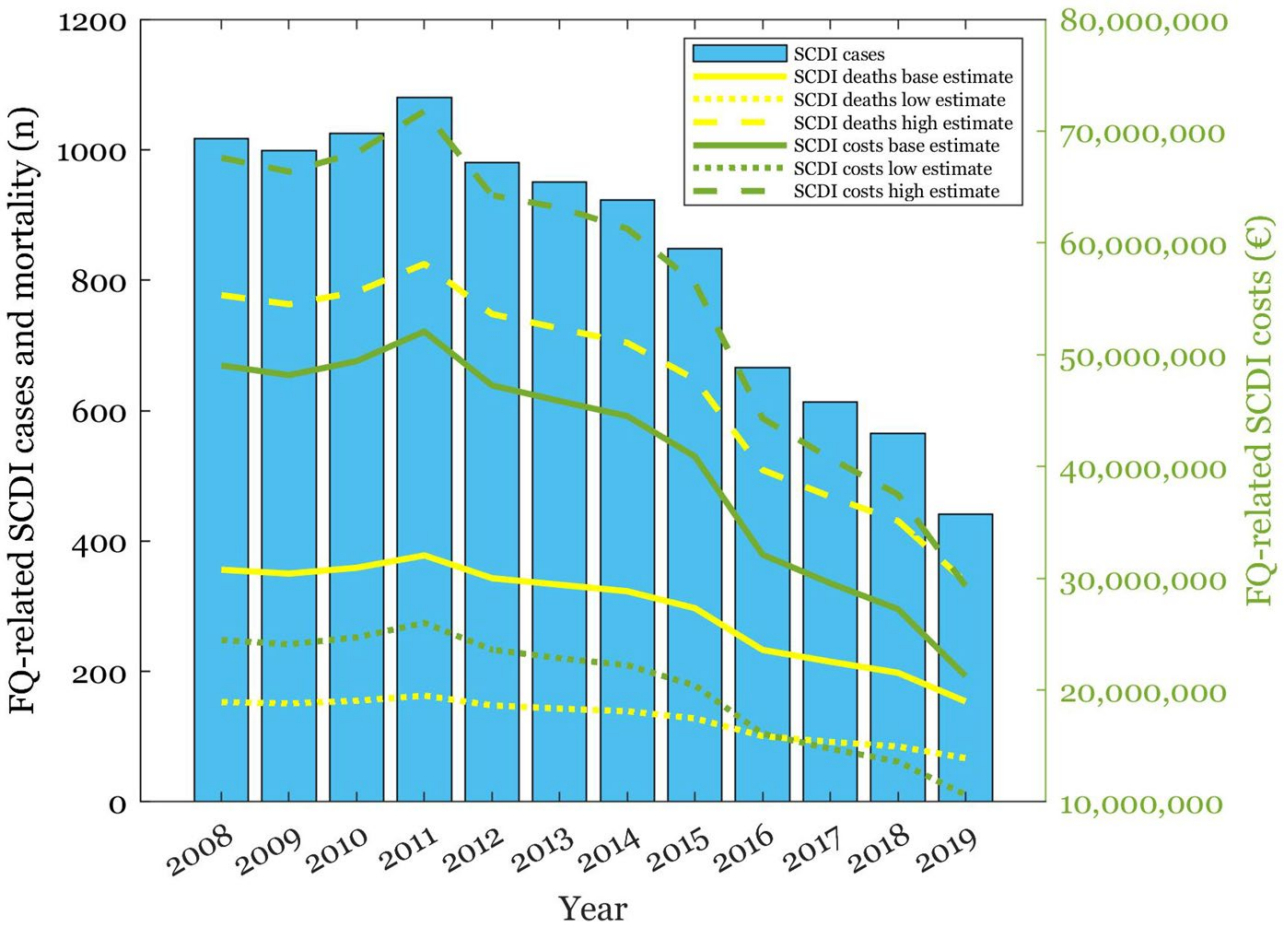
FQ‐related SCDI cases, and their mortality and healthcare costs depicted with baseline, low and high estimates

**FIGURE 4:**
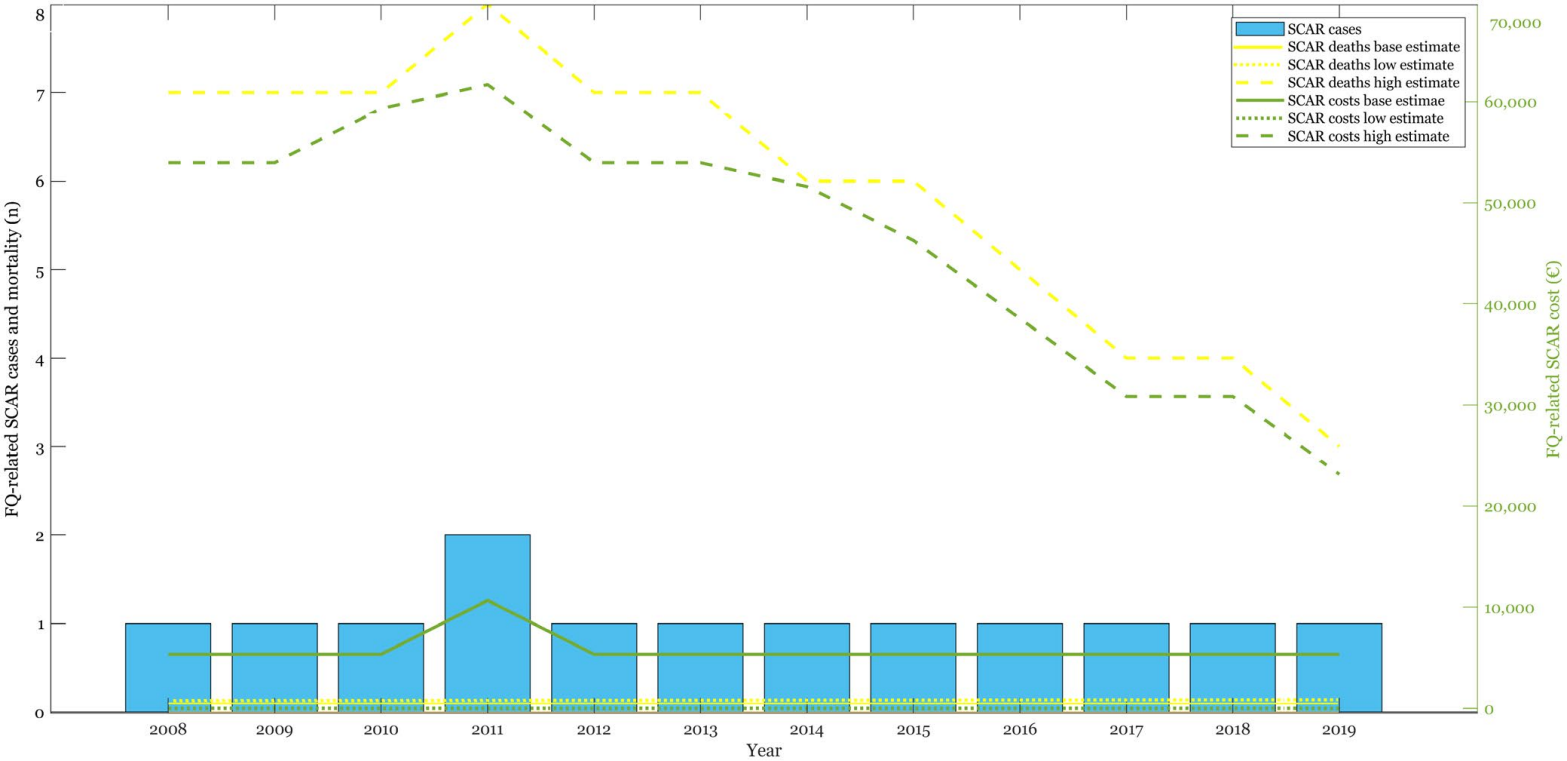
FQ‐related SCAR cases, and their mortality and healthcare costs depicted with baseline, low and high estimates

**FIGURE 5:**
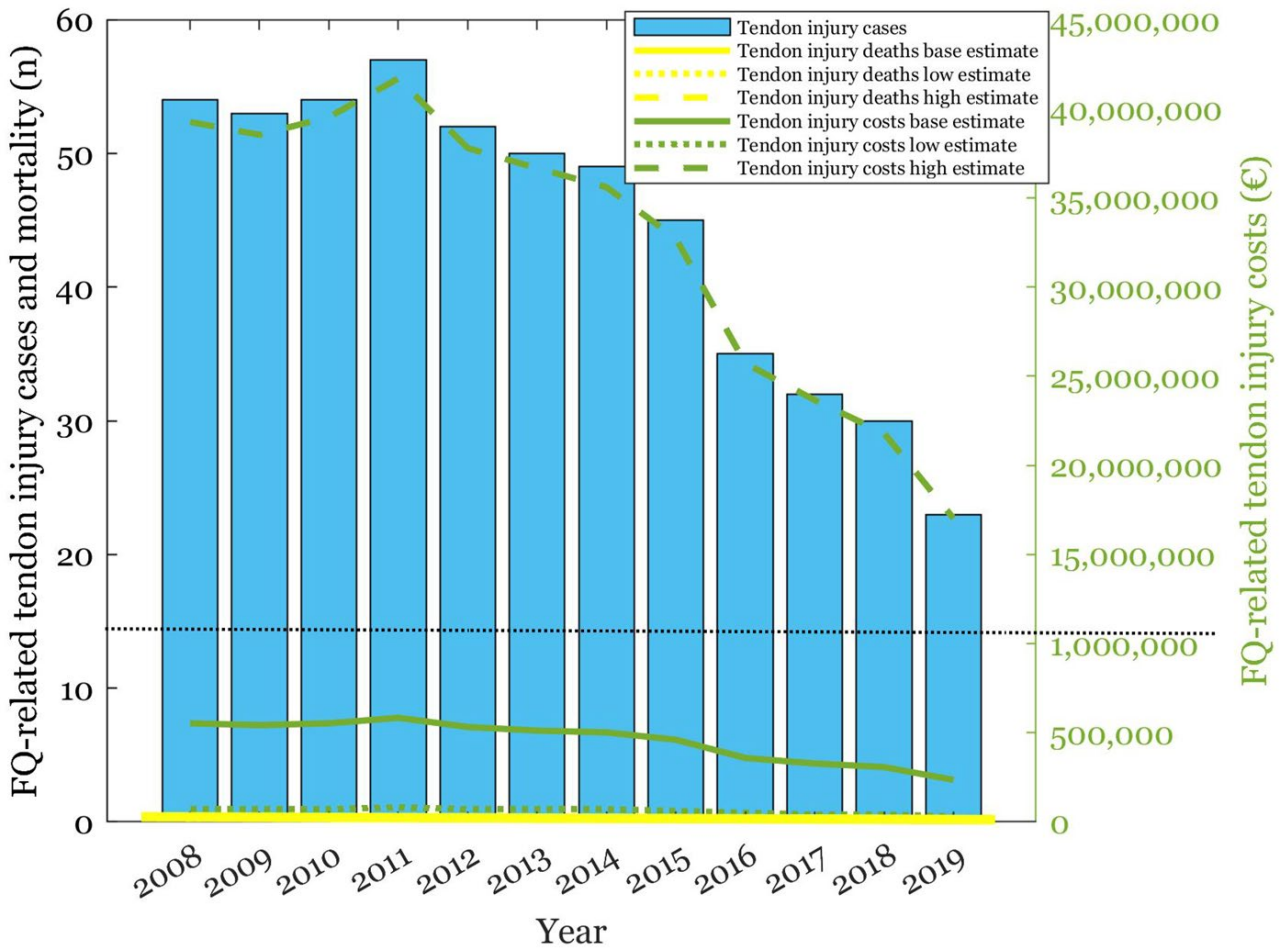
FQ‐related tendon rupture cases, and their mortality and healthcare costs depicted with baseline, low and high estimates. The costs axis is broken with a black dotted line to display the high estimate

**FIGURE 6:**
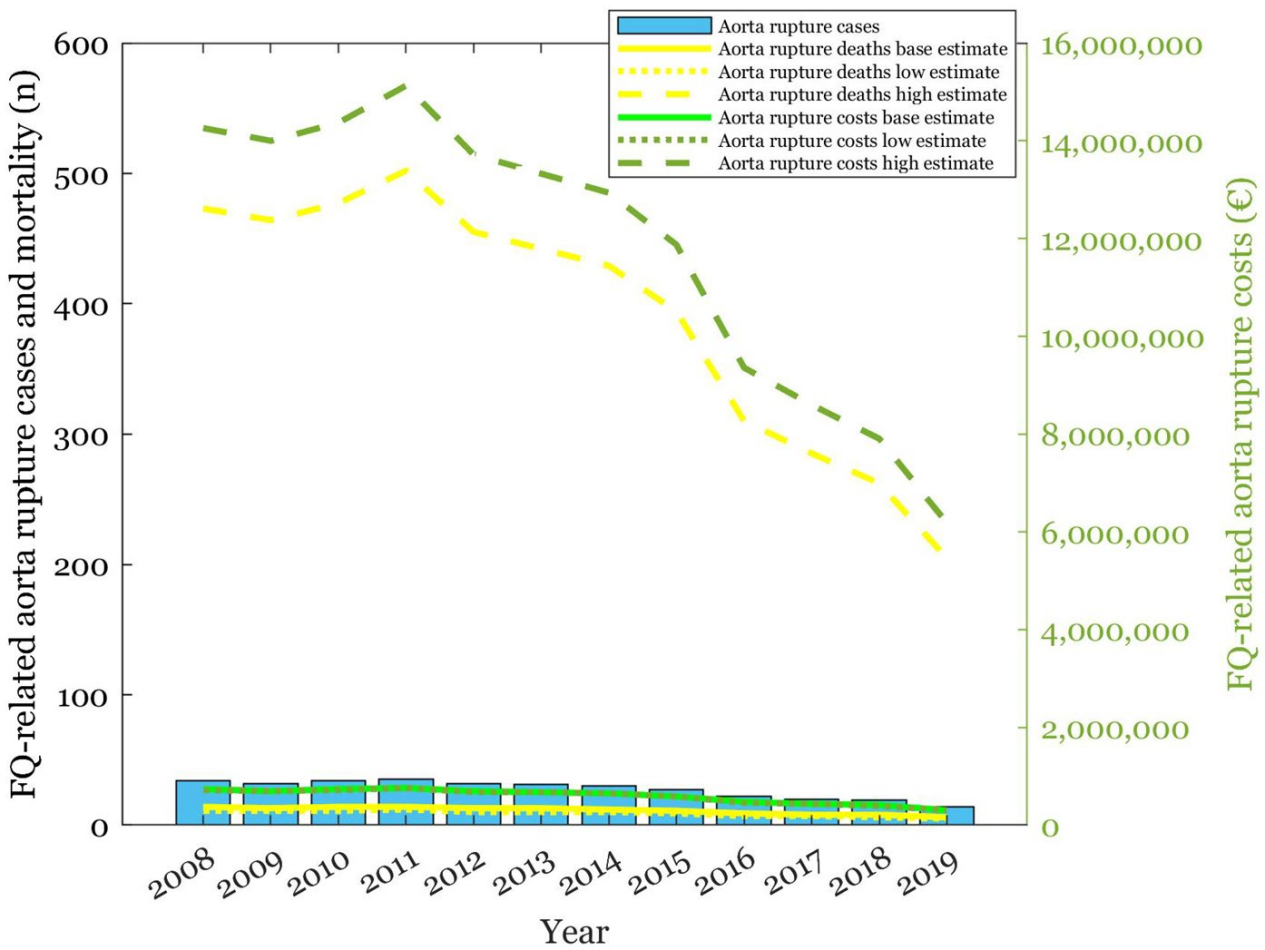
FQ‐related aortic rupture cases, and their mortality and healthcare costs depicted with baseline, low and high estimates

**FIGURE 7:**
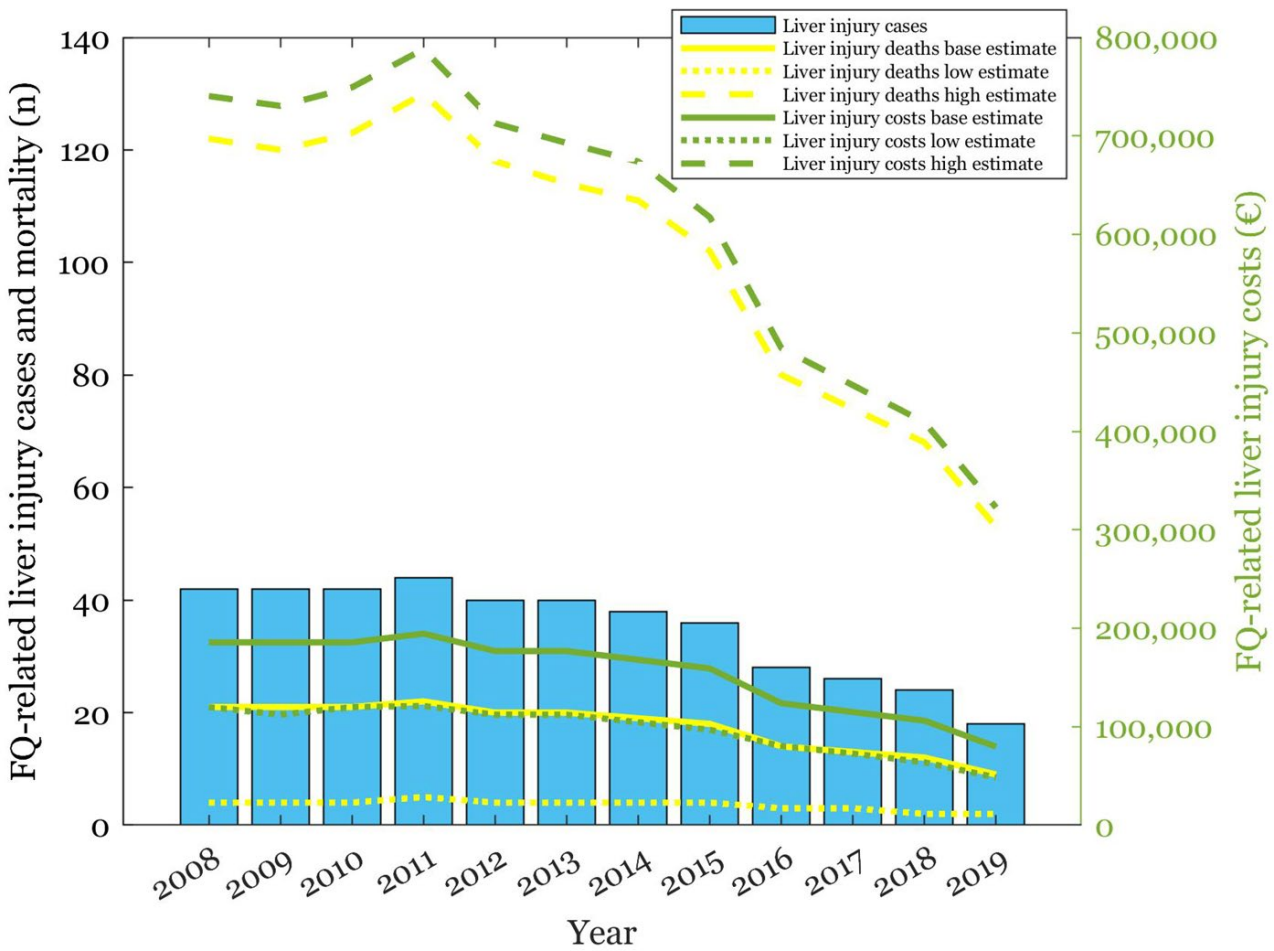
FQ‐related liver injury cases, and their mortality and healthcare costs depicted with baseline, low and high estimates

Cases, mortality, and costs associated with FQ‐related serious ADRs are dependent on the amount of FQs prescribed. The results, depicted in Figures 2, 3, 4, 5, 6, 7, and Table 2, showed that serious ADRs associated with FQs led to a total healthcare cost of 501,938,402 € during the 12‐year period according to baseline estimates. The average direct healthcare cost of a serious FQ‐related ADR was 44 010€. Each FQ prescription in Finland carried a 0.62% risk of a serious ADR indicating that cost of serious ADRs was 274€ per prescription. The estimated total number of serious FQ‐related ADRs was 11,405 between 2008 and 2019. All patients with serious ADRs were hospitalized and required follow‐up clinic visits after recovery.

**TABLE 2:** Costs and mortality associated with serious FQ‐related ADRs in 2008–2019 with sensitivity analyses

ADR type	Total ADRs, n	Cost of ADR, recovered patient (€)	Cost of ADR, dead patient (€)	Total healthcare costs (€)	Total Mortality, n	Sensitivity analysis of total healthcare costs, range (€)	Sensitivity analysis of total mortality, range, n
SCDI	10,108	46,005.00	52,324.00	487,381,481.00	3,539	243,479,890.00– 670,757,642.00	1,525– 7,718
SCAR	13	5,331.00	2,379.00	69,303.00	0	0– 558,072.00	0–71
Tendon rupture	534	10,242.00	N/A	5,469,228.00	0	737,424.00– 390,005,118.00	0 – 0
Aortic rupture	330	21,703.00	21,703.00	7,161,990.00	135	7,053,475.00– 141,633,778.00	109– 4,698
Liver injury	420	7,683.00	1,157.00	1,856,400.00	210	1,163,786.00– 7,367,763.00	43– 1,215
Totals	11,405			501,938,402.00	3,884	252,434,575.00–1,210,322,373.00	1,677–13,702

In addition to SCDIs requiring the longest stays in hospital (up to 53 days) and being the costliest FQ‐related ADRs, they were also the most frequent serious ADRs (n = 10 108). The healthcare costs of a patient, who recovered from FQ‐related SCDI were 46 005€. A patient, who did not recover from the infection, had even higher healthcare costs, i.e., 52 324€. Total costs of SCDIs amounted to 487 381 481€. Other serious FQ‐related ADRs were significantly less frequent. Aortic and tendon ruptures tallied up total costs of 7 161 990€ with 330 events and 5 469 228€ with 534 events, respectively. The total estimated mortality associated with serious FQ‐related ADRs between 2008 and 2019 was 3,884, with SCDI being the leading cause of death (n = 3,539). No deaths were associated with tendon ruptures or SCAR with the baseline estimates. FQ‐related liver injuries were associated with 210 and aortic ruptures with 135 deaths during the 12‐year observation period. According to baseline estimates, the average mortality risk associated with FQ‐related ADRs was 0.21%.

The results of the sensitivity analyses are shown in Figures 3, 4, 5, 6, 7 and Table 2. Parameter changes in incidence rates triggered large variation, especially in the worst‐case scenarios. Mortality increased most significantly in aortic ruptures and liver injuries. Distinctly higher healthcare costs occurred particularly in tendon ruptures and aortic ruptures.

**FIGURE 8:**
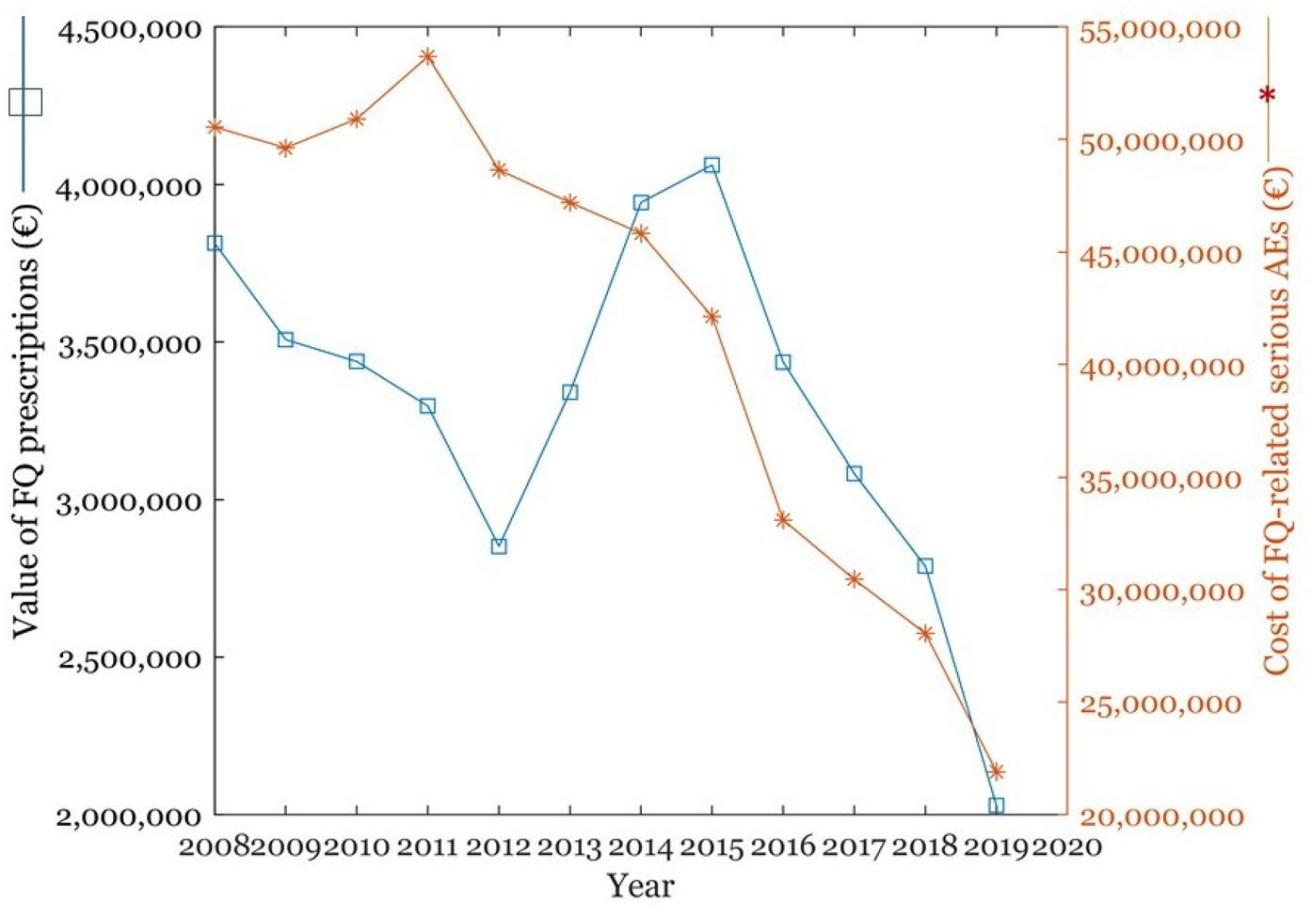
The monetary value of FQ prescriptions filled between 2008 and 2019 and the corresponding cost of FQ‐related serious ADRs

The declining number of FQ prescriptions has meant that both total medication costs and ADR costs have significantly fallen since 2008 (Figure 8). However, policy changes have also taken place to restrain rising medication costs for both patients and society. Key changes have included the implementation of generic substitution in 2003, which was complemented with the reference price system, introduced in 2009.^61^

## 4 DISCUSSION

The economic burden of ADRs is often overlooked in decision making, although healthcare resources are limited, and overall medication expenditure is rising. While ADRs remain a notable cause of morbidity and mortality, the number of ADR‐related deaths seems to have decreased in recent years.^7^ In this present study, 1 831 537 FQ prescriptions filled between 2008 and 2019 were estimated to trigger 11 405 ADRs and 3,884 deaths, resulting in a total healthcare cost of 501 938 402€ during the 12‐year observation period. On average, the direct healthcare cost of a serious FQ‐related ADR amounted to 44 010 €. FQ‐related serious ADRs were dependent on the amount of FQs prescribed, with a decline in an annual number of ADRs, mortality, and costs, consistent with the concurrent declining pattern in all antimicrobial prescribing.

ADR research relies heavily on timely spontaneous ADR‐reporting, thorough post‐marketing surveillance, and observational research. However, the true extent and nature of all drug‐induced ADRs will continue to elude healthcare professionals and decision makers if reporting levels remain poor. The importance of post‐marketing surveillance from regulatory agencies such as EMA cannot be stressed enough. Without it, there is no incentive for pharmaceutical companies to continue research after marketing authorization. Aortic ruptures and neuropsychiatric ADRs represent types of FQ‐related ADRs identified only recently, which can be difficult to distinguish from other causes but have distinct harmful clinical and economic implications. A particular challenge of the study was the lack of data concerning ADR costs. Due to this, we were unable to estimate the costs of several known FQ‐related ADRs, such as neuropsychiatric events. Therefore, although the costs and mortality presented in this study are significant, they are likely still an underestimation of the full economic impact of FQ‐related ADRs. The shortage of comprehensive cost data directed us to conduct the study from the perspective of the healthcare provider, and, therefore, direct costs borne by other parties, such as patients and employers, were not included, in addition to the loss of productivity. A societal perspective would include all costs regardless of by whom they are borne and would be ideal.

The role of pharmacogenomics is becoming more important in the ADR research. In connection to FQs, for example, studies have shown significant pharmacokinetic variability among tuberculosis patients treated with moxifloxacin, which is thought to be connected to variations in genes encoding drug‐metabolizing enzymes and drug transporters.^62^, ^63^ In the future, pharmacogenomics is expected to aid in the identification of patients, who are more likely to experience ADRs, and prevent unexpected, dose‐independent adverse reactions. Although literature sources similar to the Finnish population were favored, when sourcing risk estimates, our model did not consider pharmacogenomics related to various ADRs.

This study drew its estimates from several published sources and databases, which generated parameter uncertainty. Variability in ADR incidence estimates is caused by differences in study populations, geographical areas, and hospitals, in addition to methodological differences in classifying ADRs. Although death is the worst ADR, its precise incidence remains equally difficult to estimate due to similar challenges. Sensitivity analyses were conducted to expose the model to extreme situations. The best‐case scenario probably underestimates, and the worst‐case scenario likely overestimates cases, mortality, and costs, leaving the actual outcomes somewhere in between. The model inputs involving probabilities of serious ADRs and mortality associated with them were obtained from published literature, which enables that part of the model to be generalized and transferred to other countries. Although the full model with the current model parameters describes ADR costs in a setting that is unique to Finland, the cost inputs in the model can be modified independently and utilized to generate internationally transferable results.

Tendon ruptures are no doubt the most distinctive and familiar serious FQ‐related ADRs. However, particular emphasis should be on patient groups with an increased risk of developing CDI. SCDIs are the most frequent, fatal, and costly serious adverse reactions associated with the use of FQs, and the need for cost‐effective therapeutic alternatives will continue. According to our study, SCDIs cause more than 95% of the total direct healthcare costs of the ADRs. Although the number of FQ‐associated SCDI initially fell rapidly in Finland until 2020 along with declining use of FQs,^27^, ^64^ the general trend of CDI incidence is increasing in most countries and the impact of the on‐going COVID‐19 pandemic is concerning.^65^ According to a recently published rapid review and meta‐analysis, the global prevalence of antibiotic prescribing has been around 75% in COVID infections, while bacterial co‐infections are only present in 6%–8% of patients, suggesting a fair amount of unnecessary use.^66^ The increased use of broad‐spectrum antimicrobials is expected to add to the burden of CDI, and rates were reported to have increased from 3.32/10,000 patient‐days to 3.60/10,000 patient‐days already during early 2020.^67^ The risks and benefits of FQs should be weighed carefully also during the COVID pandemic since patients most vulnerable to FQ‐related serious ADRs are often the ones most seriously affected by the virus.

## 5 CONCLUSIONS

Although FQs continue to be generally well‐tolerated antimicrobials, serious ADRs can cause long‐term disabilities to patients and high healthcare costs. In fact, on average, FQ‐related ADRs appear to be approximately ten times more expensive than the FQ medications themselves. The most impactful of the FQ‐related ADRs are *clostridioides difficile* infections, which alone cause more than 95% of the total direct healthcare costs of the ADRs. Accordingly, from the economical perspective, efforts should be focused on limiting the risk of FQ‐associated CDI. Nevertheless, the different risks and benefits of these drugs should always be weighed carefully.

## ETHICS STATEMENT

This is a retrospective observational study with registered data, and neither an ethics approval nor patient consent was required.

## DISCLOSURE

The authors of this manuscript have no conflicts of interest to declare.

## AUTHOR CONTRIBUTION

All persons who meet authorship criteria are listed as authors. All authors took part in conceptualizing the idea, designing the study, analyzing the data, and writing the manuscript. All authors have seen and approved the final version of the manuscript.

## Data Availability

The data that support the findings of this study are available on request from the corresponding author. The data are not publicly available due to privacy restrictions.
